# A Data Set of 255,000 Randomly Selected and Manually Classified Extracted Ion Chromatograms for Evaluation of Peak Detection Methods

**DOI:** 10.3390/metabo10040162

**Published:** 2020-04-22

**Authors:** Erik Müller, Carolin Huber, Liza-Marie Beckers, Werner Brack, Martin Krauss, Tobias Schulze

**Affiliations:** 1 Department of Effect-Directed Analysis, Helmholtz Centre for Environmental Research—UFZ, Permoserstrasse 15, 04318 Leipzig, Germany; erik.mueller@ufz.de (E.M.); carolin-elisabeth.huber@ufz.de (C.H.); liza-marie.beckers@ufz.de (L.-M.B.); werner.brack@ufz.de (W.B.); martin.krauss@ufz.de (M.K.); 2 Institute of Biology V, RWTH Aachen University, Worringerweg 1, 52074 Aachen, Germany

**Keywords:** peak detection, peak picking, EIC, XIC, LC-MS

## Abstract

Non-targeted mass spectrometry (MS) has become an important method over recent years in the fields of metabolomics and environmental research. While more and more algorithms and workflows become available to process a large number of non-targeted data sets, there still exist few manually evaluated universal test data sets for refining and evaluating these methods. The first step of non-targeted screening, peak detection and refinement of it is arguably the most important step for non-targeted screening. However, the absence of a model data set makes it harder for researchers to evaluate peak detection methods. In this Data Descriptor, we provide a manually checked data set consisting of 255,000 EICs (5000 peaks randomly sampled from across 51 samples) for the evaluation on peak detection and gap-filling algorithms. The data set was created from a previous real-world study, of which a subset was used to extract and manually classify ion chromatograms by three mass spectrometry experts. The data set consists of the converted mass spectrometry files, intermediate processing files and the central file containing a table with all important information for the classified peaks.

## 1. Summary

Non-target liquid chromatography mass spectrometry (LC-MS) has gained much importance over recent years in metabolomics, environmental sciences, and other fields. In today’s biochemical studies, it becomes increasingly important to gather holistic knowledge about samples from all origins (e.g., tissue, urine, water samples). Rather than just monitoring a predetermined set of substances, which elute at specific retention times with a specific mass-to-charge ratio (*m*/*z*), non-targeted LC-MS allows researchers to detect, prioritize and identify thousands of unknown substances in their samples. The collection of ion chromatograms that reveal the substances without a priori knowledge of their identity in these samples (called peak detection) is the first and therefore an especially important step to gather as complete information as possible from the mass spectral raw data. The handling of thousands of peaks requires the availability of reliable and optimized algorithms for the sample processing. In the best case, optimization of these algorithms is based on an experimental design (DOE) and could be deployed automatically. However, only IPO [1] provides an automated optimization procedure for XCMS [2] and there are only a few studies which used DOE to optimize instrumental and software settings, such as Hu et al. [3] and Eliasson et al. [4].

Many software packages like XCMS and MZmine [5] have evaluated the performance of their software packages on whole data sets and performed well in these analyses, but there is not yet any standardized way or data set to measure the performance of peak detection algorithms. While there is a model data set available to test the performance of alignment tools [6], none such data set exists specifically for the performance testing of peak pickers and especially “gap fillers”. Gap fillers are algorithms that detect new peaks after aligning data from multiple samples by reevaluating the extracted ion chromatograms at the *m*/*z* and retention time positions in the samples where no peak was detected. The main reason is the fact that all of these well-known software packages require more information than just the extracted ion chromatograms in some of their processing steps and that these steps are only sporadically being evaluated as separated algorithms. For example, the initial step of most peak pickers—the discovery of relevant chromatogram regions—requires a holistic mass spectrometry measurement with the ion chromatogram of every scan at every *m*/*z* available.

This descriptor provides a data set that was primarily conceptualized to evaluate gap fillers that work based on EICs, but it also has a limited use in evaluating the consistency of peak pickers across different samples. To ensure the practical applicability of this evaluation data set, the data set chosen for this study were from a real scientific study that used primary, secondary, calibration and blank samples. 5000 peaks per each of the 51 samples have been automatically extracted and manually annotated by mass spectrometry experts so that this data set can be used to evaluate the quality of gap fillers and peak pickers. To make this possible, all 255,000 EICs and the presence/absence table of the peaks are provided to use as a training or evaluation data set. The data set is available in two versions: One with an automatic post-procession applied to remove additional EICs classified as peaks and one without.

## 2. Data Description

The raw and derived mass spectral files are available at MetaboLights under https://www.ebi.ac.uk/metabolights/MTBLS1455. The whole data set is available at Zenodo under https://doi.org/10.5281/zenodo.3756211. The paths described in this section are relative to the root directory of the Zenodo repository.

### 2.1. MS Data

The converted LC-MS data files in .mzML format files are available in the folder "0_MS_data/Data_files".

### 2.2. Processed Data

The results from the MZmine workflow are in the table "1_Processed_data/Original_MZmine_table/Table_1_Holtemme_Stichtag2015_water_ESIpos_MRP2.7_gap-filled.csv". The randomly selected subset that was used for the annotation are in "1_Processed_data/Selected_subset_MZmine_table/Table_2_Holtemme_Stichtag2015_water_ESIpos_MRP2.7_gap-filled_clean.csv".

EICs as an .RData file are accessible at "1_Processed_data/EIC_data/EIC_data.RData". The .RData object contains two lists called EIC\_list\_7 and EIC_list_14 with the EICs with a retention time window of ±7 and ±14 s, respectively. Each of the lists contain 51 elements named after the samples. These lists each contain a list of 5000 elements containing EICs, named after the peak ID from which they originate. Each EIC is a matrix, with the first row representing the intensities and the second row representing the retention times of the scans.

### 2.3. Classified Data

Excel tables with the plotted EICs are provided at "2_Classified_Data/Excel_tables_for_classification". The initial classification by the mass spectrometry experts is provided in the table "2_Classified_Data/Excel_tables_for_classification/Classification_before_cleanup.csv". The post-processed classification is provided in "2_Classified_Data/Excel_tables_for_classification/Classification_after_cleanup.csv".

## 3. Methods

### 3.1. Data Set

The data set used in this study is based on the data published previously by Beckers et al. [7]. This particularly prepublished real-world data set was chosen because the objective of this study is to provide a training data set for algorithms for the processing of real-world samples.

Specifically, this data set was chosen for its known consistent factors among samples—i.e., sampling the same water package at different times along a river—while also having enough different tributaries and other factors to make the samples distinguishable.

### 3.2. Sampling

The sampling was conducted at the Holtemme river (Saxony-Anhalt, Germany). The river originates in the Harz Mountains and flows into the Bode river after 47 km. Agriculture is the dominating land use in the Holtemme river catchment. Furthermore, the river passes through two medium-sized towns and receives the effluent of two wastewater treatment plants (WWTPs). The first WWTP (WWTP I) serves an area of 300 km² with about 50,000 inhabitants. The second WWTP (WWTP II) covers an urban area of 143 km² with about 36,800 inhabitants connected to the WWTP. Further technical details on the WWTPs are presented in the Appendix A (Table A1).

At 16 spots along the river, grab samples with a volume of 500 mL were taken. To guarantee relative consistency of these samples, the time at which they were taken was adjusted to the flow rate of the river to sample the same water package at all spots. More detailed information about the samples is provided in the zenodo repository. Additionally, three two-hour composite samples intervals of the WWTP effluents were provided by the operators. These samples were taken time-proportional in 2 min intervals.

### 3.3. Chemical Analysis

#### 3.3.1. Instrumentation

The analysis was performed on an UltiMate 3000 LC system (Thermo Scientific, Waltham, MA, USA) connected to a QExactive Plus mass spectrometer (Thermo Scientific) with a HESI source.

#### 3.3.2. Sample Preparation

To prepare the samples for a direct large volume injection of 100 μL, a 2 M ammonium formate buffer, 25 μL of methanol and 25 μL of an internal standard mixture containing 40 isotope-labelled compounds (40 $\frac{\mathrm{ng}}{\mathrm{mL}}$) were added to 1 mL of sample. These 40 internal standards were added to all measured samples except for the blanks.

#### 3.3.3. Chromatography

A Kinetex 2.6 μm EVO C18 (50 × 2.1 mm) column with a pre-column (C18 EVO 5 × 2.1 mm) and an inline filter was used at 40 °C to perform chromatographic separation of the samples. The solvent gradient across all samples was identical (Table A2 in Appendix B) [8].

#### 3.3.4. Mass Spectrometry

The data provided in this study was measured at the nominal resolving power of 140,000 referenced to *m*/*z* 200 in full-scan mode with a positive ionization. The sheath gas flow rate was 45, the aux gas flow rate was 1. The spray voltage was set to 3.8 kV. The capillary temperature and the aux gas heater temperature were both set to 300 °C. The S-lens RF level was 70. The scan range was set to a *m*/*z* of 100–1000. At the beginning and at the end of the batch, calibration standards were run at 1, 10, 100, and 1000 $\frac{\mathrm{ng}}{L}$ for quality control purposes (see Appendix C).

### 3.4. Data Processing

#### 3.4.1. Peak Picking and Alignment

The samples 161010_ 29_ Stich_ 24 and 161010_ 30_ Stich-25 were removed from the data set, as no complete LC run was acquired. Raw data from the LC-HRMS were converted into mzML format (centroid mode) by ProteoWizard v3.0.18265. Peak lists were generated using the software MZmine v2.26 with the following settings. Parameter descriptions were extracted from the MZmine help.

1. Mass Detection:
  (a) Noise level 5e3
2. ADAP chromatogram builder
  (a) Min group size of # of scans: 15
    - In the entire chromatogram there must be at least this number of sequential scans with points above the group intensity threshold set by the user.
  (b) Group intensity threshold: 5000
  (c) Min highest intensity: 5000
    - There must be at least one point in the chromatogram that has an intensity greater than or equal to this value.
  (d) *m*/*z* tolerance: 0.001 *m*/*z* or 7 ppm
3. Smoothing
  (a) Filter width: 7
    - This parameter sets the intensity of the smoothing effect, a higher width means a more extreme smoothing.
4. Chromatogram deconvolution
  (a) Algorithm: Local minimum search
  (b) Chromatographic threshold: 80.0%
    - Threshold for removing noise. The algorithm finds the intensity such that the specified percentage of the chromatogram’s data points are below that intensity; all such data points are removed.
  (c) Search minimum in RT range (min): 0.15
    - A local minimum is considered to be the point that separates two adjacent peaks if it is minimal in the specified retention time range.
  (d) Minimum relative height: 20.0%
    - Minimum height of a peak relative to the chromatogram’s highest data point.
  (e) Minimum absolute height: 5.0 × $10^{4}$
    - Minimum absolute height of a peak for it to be recognized.
  (f) Min ratio of peak top/edge: 2.7
    - Minimum ratio between a peak’s top intensity and side (lowest) data points. This parameter helps to reduce the detection of false peaks in cases where the chromatogram is not smooth.
  (g) Peak duration range (min): 0.15–4.00
    - Range of acceptable peak durations
5. Alignment
  (a) *m*/*z* tolerance: 0.001 or 7 ppm
  (b) Weight for *m*/*z*: 80
  (c) Retention time tolerance: 0.3 absolute (min)
  (d) Weight for RT: 30

The files were entered into MZmine in lexicographical order. The resulting peak list with 32,611 aligned peaks was then exported into a table in .csv format, which was then further processed.

#### 3.4.2. Extraction of EICs

5000 peaks out of the 32,611 were chosen at random using the sample function from R version 3.6.1. [9] with the random seed 4627. The corresponding *m*/*z* and retention time for each of the 5000 peaks were extracted and then used to extract ion chromatograms across all samples at these positions, resulting in 255,000 (51×5000) EICs. For peaks that were picked by the peak picker, the retention time of the specific peak in the specific sample were used instead of average retention time. The EICs were extracted using the rawEIC function from the XCMS package with a ppm of 3.5 and a retention time window of ±7 s. For each of the EICs, a weighted mean (with the intensities as weights) of the retention time was calculated to find the center retention time of the peak, if present. At these centered peak retention times, new EICs were extracted, two for each peak with a ppm of 3.5 and a retention time window of ±7 and ±14 s, respectively. These EICs were then stored in .RData objects.

This recalculation of the retention times for each peak in each sample is necessary due to the naturally occurring small retention time shifts in LC-MS experiments. It is also easier and clearer for researchers to determine whether the EIC contained a peak when the central position in the chromatogram was also the weighted center of the intensities within that chromatogram. Furthermore, if there was just random electronic noise in that region of the chromatogram, shifting the retention time would not have a large impact on the resulting EICs.

### 3.5. Classification

#### 3.5.1. Initial Classification

**Figure 1:**
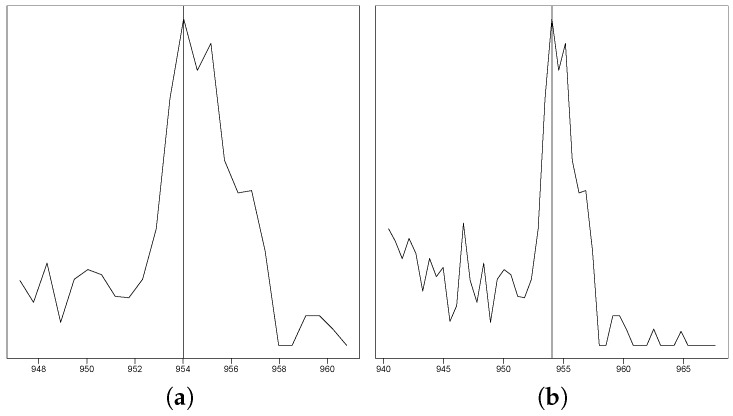
This figure exemplarily shows one out of the 255,000 peak plots that the experts evaluated. (**a**) The EIC in a ±7 s retention time window. (**b**) The EIC in a ±14 s retention time window.

The EICs with the different retention time windows for each peak were plotted side by side and inserted into 51 excel files using the R package openxlsx version 4.1.0.1 [10], with one file per sample and each file containing 5000 EICs. Each EIC pair was inserted in such a way that there was one EIC pair per excel row. By using this format, it was easier to quickly evaluate the EICs at a glance. The excel files were split up among 3 mass spectrometry experts, who went through the EICs one by one and marked them with either a 1 to denote when an EIC pair definitely does not represents a peak, a 0 to denote when the information in the EIC pair is not conclusive enough and left empty to denote when the EIC pair definitely showed a peak. The experts only annotated peaks by their shape of the intensity curve, as seen in Figure 1. This led to several nonpeak EICs being included as peaks, such as very low-intensity curves or peaks that are supposedly only present for a single scan. This was a deliberate decision, only keeping easily removable non-peaks in the data set and making the data set consistent for gap fillers that work based purely on peak shape. This data set was stored in its original excel format and saved by each researcher. To make it more accessible, it was then re-read using the R package readxl [11] and aggregated into a csv table. This is the first data set without post-processing, and the result is provided in "2_Classified_Data/Excel_tables_for_classification/Classification_before_cleanup.csv" in the Zenodo repository. A more in-depth analysis of the results of the expert classification process is available in Appendix D.

#### 3.5.2. Automated Post-Processing

There is also a version of the data set available with a very simple post-processing applied to mitigate the drawbacks of the manual evaluation. To clean up the classified peaklist, three transformations on the peaklist have been applied:

1. All single-length scans have been removed from the EICs. This means that every scan that has a zero-intensity scan before and after it has also been set to zero. This was done over all scans in all EICs, because (especially at low intensities) it has been observed that often that a random uptick of electronical noise caused single-scan peaks.
2. All peaks with a scanlength of 2 or smaller have been removed if the sum of their intensities was lower than $1\times 10^{5}$.
3. All peaks with a sum lower than $1\times 10^{4}$ have been removed.

These criteria are extremely conservative, yet they removed around 30,000 additional peaks. The removal of single-length and double-length scans had the highest impact on this removal. Setting the minimum number of allowed scans for peaks to a higher number or increasing the intensity threshold would make the result less conservative and would possibly exclude more EICs that experts would correctly identify as peaks. The post-processed data set is provided at "2_Classified_Data/Excel_tables_for_classification/Classification_after_cleanup.csv" in the Zenodo repository.

## 4. User Notes

The only requirements for testing and evaluation of EIC-based peak detection algorithms are the EIC list and one of the classification tables. The MS data files are supplied in the mzML format, so that users can either check the quality of their peak picking or gap-filling tools if their algorithm needs a more holistic data structure than just EICs. This data set will be provided in the initial and post-processed variants, mainly due to the fact that some trend detection algorithms might classify certain EICs as peaks because the shape implies there to realistically be one. These peaks can only be discounted by denoising or setting an intensity cutoff, which might be a separated from the detection or filling algorithm.

## Figures and Tables

**Figure A1:**
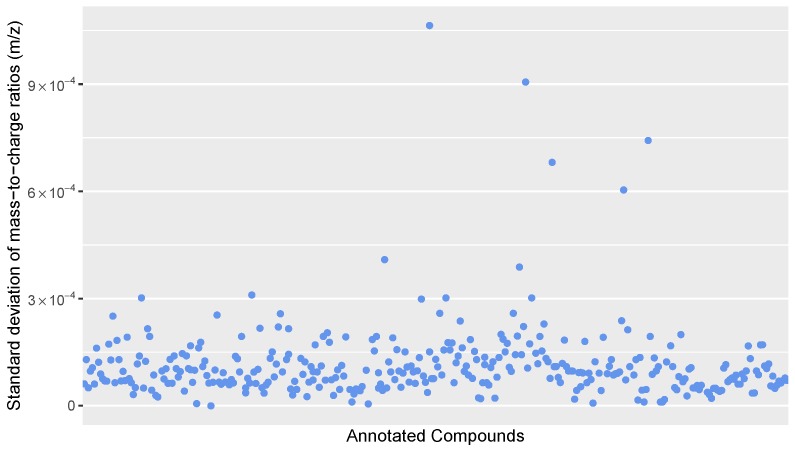
Scatter plot of the standard deviation of mass-to-charge ratios for all annotated compounds in the calibration standards.

**Figure A2:**
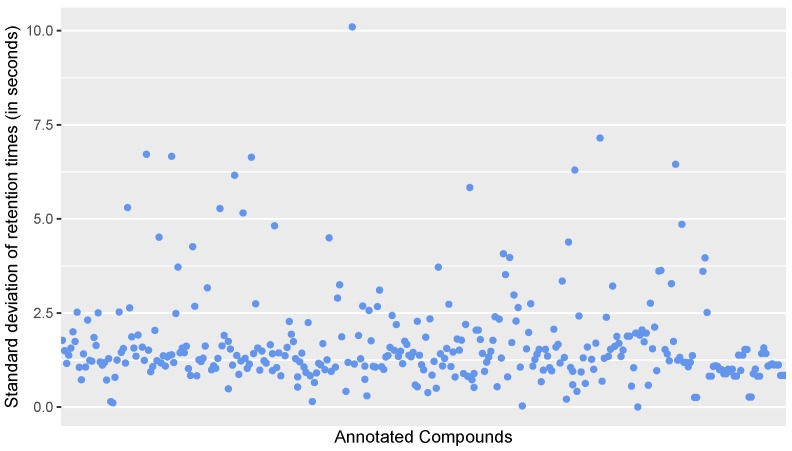
Scatter plot of the standard deviation of retention times in seconds for all annotated compounds in the calibration standards.

**Figure A3:**
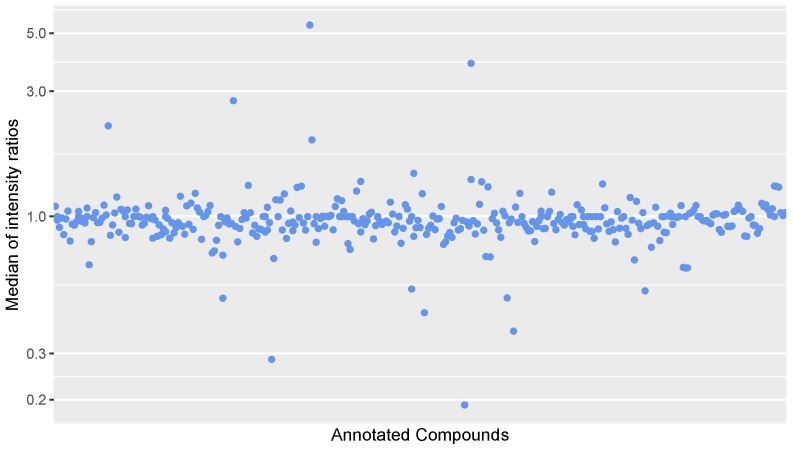
Scatter plot of the median of intensity ratios for each concentration level of annotated compounds in the calibration samples. The intensity ratios were calculated by dividing the intensity in the second calibration sample at a level by the intensity in the first calibration sample of that same level.

**Figure A4:**
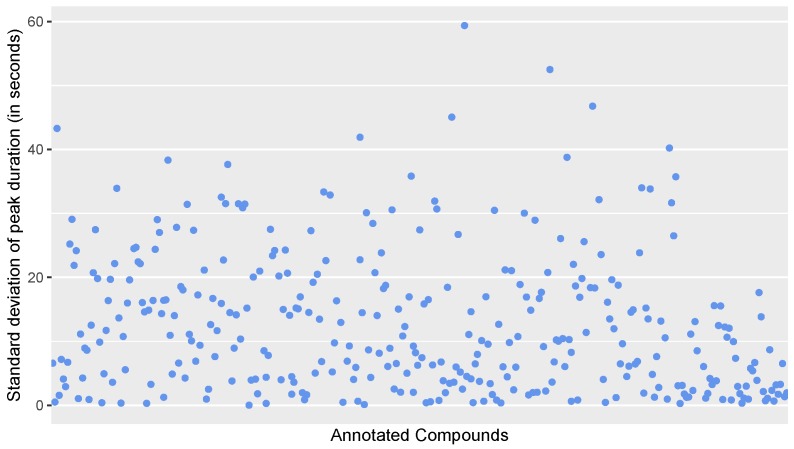
Scatter plot of the standard deviation of peak durations in seconds for all annotated compounds in the calibration standards.

**Figure A5:**
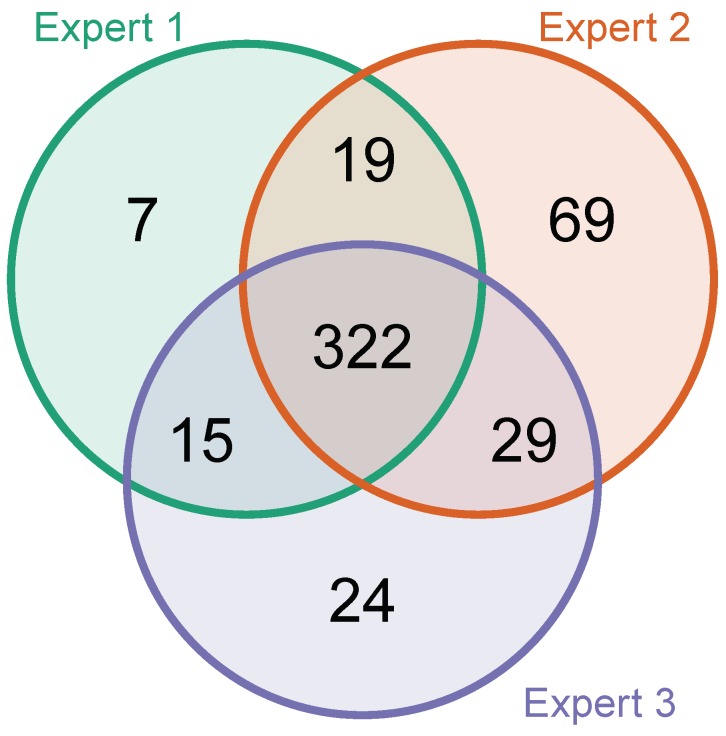
A Venn diagram of agreement of classification among experts. This graphic shows how many and which EICs were classified as "positive" and how often the experts partly or completely agreed upon this classification. EICs that have been classified as "inconclusive" have been left out.

**Figure A6:**
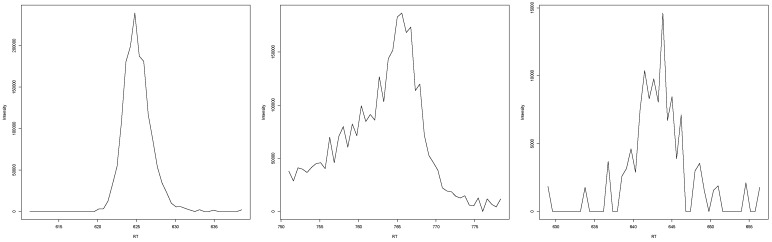
Three examples of EICs that were classified as peaks by all experts.

**Figure A7:**
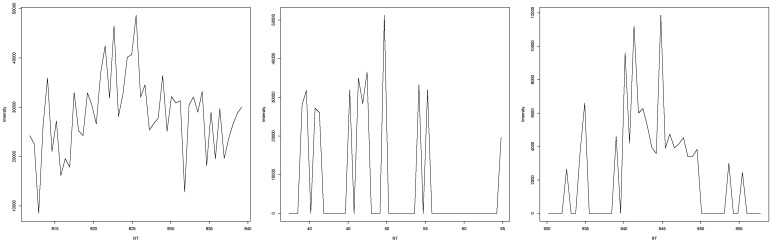
Three examples of EICs that were classified as "not peaks" by all experts.

**Figure A8:**
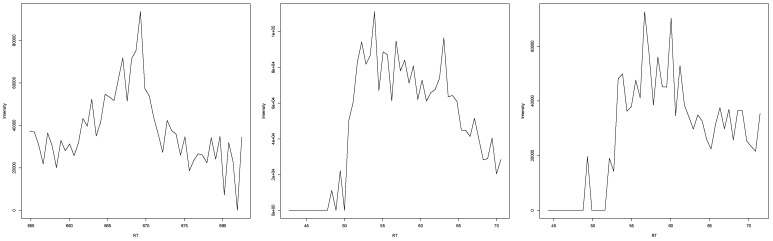
Three examples of EICs where experts either disagreed or were uncertain about them representing a mass spectrometric peak.

## Abbreviations

The following abbreviations are used in this manuscript:

LC-MS	liquid chromatography mass spectrometry
EIC	extracted ion chromatogram
*m*/*z*	mass-to-charge ratio
WWTP	waste water treatment plant
HESI	heated electrospray ionization

## Appendix A. WWTP Information

**Table A1:** WWTP Information of the Holtemme River.

	WWTP I	WWTP II
**Sewer system**	Separate sewer system	Combined sewer system
**Treatment technology**	Activated sludge	Activated sludge
**Connection rate [%]**	98	99.4
**Size (population equivalents)**	80,000	60,000
**Connected inhabitants**	50,000	36,800
**Connected industry (population equivalents)**	15,000	5400
**Hydraulic retention time [h]**	ca. 72	54 (dry weather ∼7500 m^3^) 15 (rain event ∼27.000 m^3^)
**Daily discharge [m^3^] on sampling day**	10,042	6617
**Temperature of effluent water [°C] on sampling day**	16.7	18.01

## Appendix B. Solvent Gradient Information

**Table A2:** Solvent gradient for liquid chromatography.

Time [min]	Flow Rate [mL]	Solvent A [%] Water + 0.1% Formic Acid	Solvent B [%] Methanol + 0.1% Formic Acid	Solvent C [%] Acetone/Isopropanol (50:50)
0	0.3	95	5	0
1	0.3	95	5	0
13	0.3	0	100	0
24	0.3	0	100	0
24.1	0.35	5	10	85
26.2	0.35	5	10	85
26.3	0.35	95	5	0
31.9	0.35	95	5	0
32	0.3	95	5	0

## Appendix C. Evaluation of Calibration Standards

We evaluated the calibration files at the beginning and the end of the run in order to verify that **(1)** mass-to-charge ratios, **(2)** retention times, **(3)** intensities and **(4)** peak durations stay consistent for the duration of the run. Since the MZmine gap-filling sometimes aligns heavy outliers, we used only the peak picked data for this part of the evaluation.

Figure A1, Figure A2, Figure A3 and Figure A4 show the evaluation for these measures. All figures show that the evaluated measures are consistent for the calibrations substances across the calibration standards.

## Appendix D. Peak Quality Assessment

### Appendix D.1. In-Depth Analysis of the Expert Classification of Peak Quality

1071 additional EICs (21 per file) were categorized by each expert to understand if there are common disagreements about what constitutes a mass spectrometric peak. The Venn diagram in Figure A5 shows that there were few disagreements on whether EICs represent a peak.

Table A3 contains additional information on how often the experts voted differently on the EICs in the data set. Out of the 1071 classified EICs, experts disagreed strongly (i.e., one or more experts voted “True” and one or more voted “False” on the same EIC) in 223 of the cases. Total agreement among experts with all three giving the same vote was reached in more than half (608) of the EICs. 240 of the EICs were classified without any strict disagreement, with one or more experts classifying the EIC as "unclear" and one or more experts classifying the EIC together as either “True” or “False”.

**Table A3:** Expert agreement on EIC classification

All True	All False	All Unclear	True, False	True, Unclear	False, Unclear	True, False, Unclear
322	285	1	163	136	104	60

### Appendix D.2. Guidelines and Examples for the Peak Quality Assessment

The initial classification of peaks was based only on the subjective knowledge of each expert in regards to previous targeted analysis. While this classification without any established guidelines might lead to disagreements, it reflects the uncertain nature of peak classification, depending on the person viewing the EIC. This subsection gives examples and shows where and how the experts agreed or disagreed on the classification of certain EICs.

In the case that EICs were classified by all experts as peaks (examples in Figure A6), we determined the shape to be almost always Lorentzian-like. While some minor noise may be present in these EICs, the underlying curve shows a clear general increasement of intensity followed by a clear general decrease with low or no surrounding noise.

In the case that EICs were classified by all experts as “not peaks” (examples in Figure A7), either no Lorentzian shape was visible or the Lorentzian shape was surrounded by very high noise, making it likely that the shape occurred randomly and not due to the elution of one specific substance.

In the case that experts disagreed or were unclear about an EIC representing a peak (examples in Figure A8), an increase and decrease in intensity were clearly visible, but the surrounding intensities made it unclear whether this was due to random occurrence or due to a substance eluting at that time. In most cases, either the noise was too high to make a clear assessment, or the increase/decrease was not as high or low as in clear peaks. It seems that a low signal-to-baseline/signal-to-noise ratio made it harder for experts to independently agree, with some judging the EIC more conservatively. Another reoccurring problem was the extraction width of the EIC; while it seemed clear in a window of 7 s that there is no peak visible in the EIC, the wider context given by the 14 s window made it seem like there was a peak present. This resulted in some judgements resulting in “unclear” rather than a clear yes or no.
